# Quantitative Phase Imaging as Sensitive Screening Method for Nanoparticle-Induced Cytotoxicity Assessment

**DOI:** 10.3390/cells13080697

**Published:** 2024-04-17

**Authors:** Anne Marzi, Kai Moritz Eder, Álvaro Barroso, Björn Kemper, Jürgen Schnekenburger

**Affiliations:** Biomedical Technology Center, University of Muenster, Mendelstraße 17, D-48149 Muenster, Germany; kai.moritz.eder@gmail.com (K.M.E.); alvaro.barroso@uni-muenster.de (Á.B.); bkemper@uni-muenster.de (B.K.)

**Keywords:** quantitative phase imaging, digital holographic microscopy, label-free cytotoxicity testing, nanoparticles, in vitro

## Abstract

The assessment of nanoparticle cytotoxicity is challenging due to the lack of customized and standardized guidelines for nanoparticle testing. Nanoparticles, with their unique properties, can interfere with biochemical test methods, so multiple tests are required to fully assess their cellular effects. For a more reliable and comprehensive assessment, it is therefore imperative to include methods in nanoparticle testing routines that are not affected by particles and allow for the efficient integration of additional molecular techniques into the workflow. Digital holographic microscopy (DHM), an interferometric variant of quantitative phase imaging (QPI), has been demonstrated as a promising method for the label-free assessment of the cytotoxic potential of nanoparticles. Due to minimal interactions with the sample, DHM allows for further downstream analyses. In this study, we investigated the capabilities of DHM in a multimodal approach to assess cytotoxicity by directly comparing DHM-detected effects on the same cell population with two downstream biochemical assays. Therefore, the dry mass increase in RAW 264.7 macrophages and NIH-3T3 fibroblast populations measured by quantitative DHM phase contrast after incubation with poly(alkyl cyanoacrylate) nanoparticles for 24 h was compared to the cytotoxic control digitonin, and cell culture medium control. Viability was then determined using a metabolic activity assay (WST-8). Moreover, to determine cell death, supernatants were analyzed for the release of the enzyme lactate dehydrogenase (LDH assay). In a comparative analysis, in which the average half-maximal effective concentration (EC_50_) of the nanocarriers on the cells was determined, DHM was more sensitive to the effect of the nanoparticles on the used cell lines compared to the biochemical assays.

## 1. Introduction

Driven by their unique physical and chemical properties, the number of newly designed nanoparticles with different shapes, sizes, materials and surface coatings is constantly increasing, as their use in commercial and medical applications [1,2,3]. However, this development has also led to concerns about their environmental and human health impacts [3]. As the demand for alternatives to animal testing is growing, suitable in vitro methods for the risk assessment of nanomaterials are of great importance [4]. The health effects of chemicals are assessed by various in vitro tests, which are partly standardized and suggested by the Organization for Economic Co-operation and Development (OECD) test guidelines [5]. Their small size but larger surface area compared to bulk materials, their high catalytic reactivity and their optical properties often make it complicated to apply established in vitro approaches for nanomaterial risk assessment [6]. This is reflected by a large number of nanotoxicological studies with often varying results [7]. One reason for the discrepancies arises from the often incompatibility of assay types with the tested nanomaterials, which is caused by the nanoparticles interference with the test system [8]. Commonly used cytotoxicity assays assess the number of metabolically active cells by enzymatically converting (tetrazolium) salts into colored (formazan) variants (MTT (3-(4,5-dimethylthiazol-2-yl)-2,5-diphenyltetrazolium bromide) [9], MTS (3-(4,5-dimethylthiazol-2-yl)-5-(3-carboxymethoxyphenyl)-2-(4-sulfophenyl)-2H-tetrazolium) [10], WST-1 and WST-8 ((2-(2-methoxy-4-nitrophenyl)-3-(4-nitrophenyl)-5-(2,4-disulfophenyl)-2H-tetrazolium)) [11], alamar blue assay [12]). Other common test methods are based on the integrity of the cell membrane, as its damage is a characteristic feature of apoptotic and necrotic cells. Neutral red [13], calcein-acetomethoxy (calcein AM) [14] and Trypan blue [15] assays detect membrane damage by observing alterations in the dye uptake of the cells [16]. Lactate dehydrogenase (LDH) assays [17] are based on the enzyme LDH being released into the cell culture supernatant when the cell membrane is damaged, and the activity of the enzyme is quantified by the reduction of a tetrazolium salt [18]. Other widely used test methods include DNA content assays, which are based on DNA staining and provide information about the number of cells [8] and DNA damage in individual cells [19]. In addition, reactive oxidative species (ROS) are measured to determine the cytotoxicity of nanoparticles in order to determine oxidative stress [15]. For ROS detection, fluorescent dyes such as dichlorofluorescein (DCF) are often used [20]. Finally, pro-inflammatory reactions are also measured for toxicity analysis using enzyme-linked immunosorbent assays (ELISA), where, usually, absorption and fluorescence are measured [15]. Many of the aforementioned commonly used test systems are based on colorimetric or fluorescence-based optical readouts of converted substrates. Nanomaterials with a high adsorption capacity and reactivity and optical properties, such as light absorption, can interfere with the readout system and, thus, cause misleading results [21]. Therefore, it is essential to establish new methods that are not sensitive to interactions with the tested nanomaterial and to find robust test procedures that combine and integrate different toxicity endpoints in a single workflow to enable the reliable and fast screening of nanoparticles [6].

Digital holographic microscopy (DHM), [22] an interferometric variant of quantitative phase imaging (QPI) [23], has been demonstrated as a feasible label-free method for the toxicity testing of, e.g., HgCl_2_ [24], cadmium [25], ricin and abrin [26], the anti-cancer drug etoposide [27] and for the assessment of the cytotoxic potential of nanoparticles by determining the cellular dry mass of native cell populations [28]. Compared with colorimetric in vitro toxicity assays that can interfere with nanomaterials, label- and dye-free DHM minimizes interactions with the sample. Moreover, due to low intensities of the illuminating laser light, the analyzed cell cultures remain in a native state, which makes the samples available for further downstream analysis.

**Figure 1:**
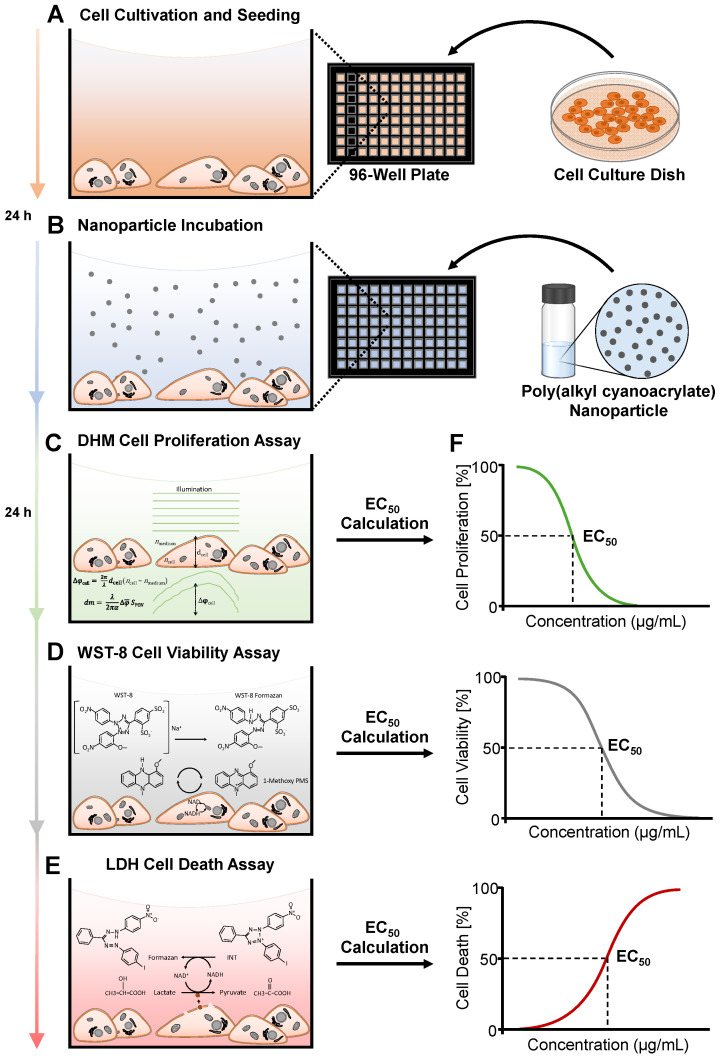
Experimental design and workflow for comparison of PACA nanoparticle in vitro cytotoxicity assessment by DHM with downstream WST-8 and LDH assays. (**A**) Seeding of NIH-3T3 and RAW 264.7 cells into 96-well plates. (**B**) Incubation of cells with PACA, cbz-loaded PACA nanoparticles and controls. (**C**) Label-free DHM QPI proliferation assay. (**D**) WST-8 cell viability assay. (**E**) LDH cell death assay. (**F**) Determination of EC_50_ values.

In this context, we investigated the capabilities of DHM in a multimodal assessment approach together with downstream biochemical assays for the cytotoxicity assessment of medically relevant nanomaterials, focusing exemplarily on polymeric nanoparticles developed for drug delivery purposes [29,30,31]. When performing three different cytotoxicity assays with the same cell populations, it is crucial that two of the assays do not irreversibly affect the investigated sample. Therefore, after the conduction of the label-free DHM assay, the removed supernatant of the cells was used for the LDH assay, while the remaining cells in the plate were analyzed in parallel by a WST-8 assay. For the reproducible and comprehensive analysis of both methodological aspects as well as particle effects, in our study, we used the well characterized commercially available RAW 264.7 macrophages and NIH-3T3 fibroblasts, as these cell lines are representative of different organs and functions, uptake and toxicity mechanisms, are frequently utilized in the cytotoxicity testing of nanomaterials and are highly compatible with DHM QPI measurements [28]. Digitonin is commonly used as positive control as it causes significant and reproducible damage due to its lytic effects on cell membranes and also because of its impact on metabolic activity and cell proliferation [32], which is consistent with the measured endpoints in the toxicity testing of nanomaterials, and it was thus chosen to validate the proper operation of the DHM, WST-8 and LDH assays. Figure 1 illustrates an overview of the experimental study design. Mouse RAW 264.7 macrophages and NIH-3T3 fibroblasts were seeded in 96-well plates (Figure 1A), incubated with unloaded and cabazitaxel (cbz)-loaded poly(alkyl cyanoacrylate) nanoparticles (PACA) (Figure 1B) and analyzed with DHM and then with the two biochemical assays. First, the cytotoxic potential was assessed with DHM-based QPI by measuring the dry mass increments of cell populations (Figure 1C). Subsequently, the viability of the same cell populations was determined with the WST-8 metabolic activity assay (Figure 1D), and the supernatants were analyzed separately in parallel for the release of LDH (LDH assay) to detect cell death (Figure 1E). The average half-maximal effective concentration (EC_50_ [33,34]) of the polymeric nanocarriers was determined (Figure 1F) to compare the performance and sensitivity of the label-free DHM with the biochemical assays and to analyze the correlation of the different methods.

## 2. Materials and Methods

### 2.1. Cell Lines and Cell Cultivation

RAW 264.7 mouse macrophages (ATCC^®^ TIB-71TM, American Type Culture Collection, Manassas, VA, USA) and NIH-3T3 mouse fibroblasts (ATCC^®^ CRL-1658TM) were cultivated according to standard cell culture procedures in Dulbecco’s Modified Eagle Medium (DMEM, Sigma Aldrich, St. Louis, MO, USA). The DMEM was supplemented with 10% fetal calf serum (FCS, PAN Biotech, Aidenbach, Germany), 1 mM pyruvate (Biochrom, Berlin, Germany), and 2 mM glutamine (Merck, Darmstadt, Germany) without antibiotics [35]. The NIH-3T3 cells were passaged three times a week, and the RAW 264.7 macrophages were passaged twice a week. Mycoplasma contamination was controlled frequently by a commercial qPCR kit (Sartorius, Göttingen, Germany). Cell culture passages 5–30 were used for experiments.

### 2.2. Nanomaterials

The tested nanoparticles, provided by SINTEF Industries (Trondheim, Norway), were PACA particles [29], which were developed for drug delivery, and cabazitaxel (cbz)-loaded PACA nanoparticles [30,31] for cancer treatment. The average particle diameter determined by dynamic light scattering (DLS) was 128 nm for the unloaded PACA nanoparticles and 170 nm, with a size distribution (polydispersity index (PDI)) ≤ 0.19, for the cbz-loaded particles. Both particles displayed a slightly negative charge with a zeta potential of about −5.19 mV (PACA) and −4.71 mV (PACA cbz). The particles were synthetized by emulsion polymerization from a water phase containing alkyl cyanoacrylate monomers and an aqueous phase containing hydrochloric acid and PEG surfactants.

### 2.3. Cell Preparation for Multimodal Cytotoxicity Assessment Experiments

RAW 264.7 macrophages and NIH-3T3 fibroblasts were cultivated up to 90% confluence and harvested with trypsin/EDTA (Sigma Aldrich, St. Louis, MO, USA). Afterwards, the cells were pelleted at 330× *g* for 5 min and resuspended into sterile filtered cell culture medium. A total of 50,000 cells/mL of NIH-3T3 fibroblasts and 150,000 cells/mL of RAW 264.7 macrophages were seeded in a volume of 300 µL into black 96-well imaging plates (µ-Plate 96 Well Black, ibidi, Munich, Germany). Cell densities were automatically determined with a label-free digital holography-based device (Fluidlab R-300, Anvajo, Dresden, Germany). The cells were incubated for 24 h at 37 °C and with 5% CO_2_ before nanoparticle treatment (Figure 1A). Therefore, different particle concentrations and a cytotoxicity control, digitonin, were prepared in freshly filtered cell culture medium. Digitonin (Sigma Aldrich, St. Louis, MO, USA) was applied at a concentration of 75 µg/mL. Unloaded PACA nanoparticles were tested at concentrations of 2–512 µg/mL for both cell lines, while cbz-loaded PACA particles were applied at concentrations of 0.002–512 µg/mL (Figure 1B).

### 2.4. Experimental Workflow of DHM with Downstream WST-8 and LDH Assays

To analyze the nanomaterial-induced effects on cell populations and to directly compare the capabilities of DHM-based QPI with the biochemical assays, DHM was measured dynamically, and for WST-8 and LDH, endpoints were determined. Initially, 96-well plates were transferred into a stage-top preheated incubator chamber on the DHM system and to perform the label-free DHM QPI proliferation assay (Figure 1C). Subsequently the 96-well plate was removed from the heating chamber, and 50 µL of the supernatant was carefully transferred to an uncoated 96-well plate with a transparent bottom. The WST-8 assay was applied to investigate the cell viability of the same cell populations (Figure 1D), while the transferred supernatant of the cells was analyzed in parallel for LDH enzyme release (LDH assay, Figure 1E) to determine cell death. Three independent experiments were performed (*n* = 3).

### 2.5. DHM QPI Cell Proliferation Assay

Cell proliferation was analyzed with DHM-based QPI as described previously [28] by the utilization of an inverted Nikon Ts2R microscope (Nikon, Tokyo, Japan) with an attached off-axis DHM module [36], a motorized microscope stage (Märzhäuser, Wetzlar, Germany) and a stage-top incubator chamber with a 5% CO_2_ atmosphere and a physiological temperature of 37 °C (K-frame heated chamber H301, Okolab, Ottaviano, NA, Italy). DHM time-lapse imaging for the quantification of nanoparticle-induced cytotoxicity was performed with a 20× microscope objective (Nikon Plan 20×/0.4, Nikon, Japan) and a fiber-coupled solid-state laser (Cobolt 06-DPL, λ = 532 nm, Cobolt AB, Solna, Sweden). Therefore, utilizing custom-built software implemented in Java and Python 3, for each investigated field of view (FOV), one bright-field image and ten holograms were captured, while the object illumination wave was modulated by an electrically tunable lens (ETL) [28] (Figure 1C). For each nanoparticle concentration and control, two FOVs in four wells (*n* = 8) were observed every 30 min over at time frame of 24 h. Time point t = 0 h was the start of the DHM measurement. Depending on the position in the 96-well plate, the DHM measurement started about 30–60 min after the nanoparticle incubation. As explained in detail in Eder et al. [28], digitally captured off-axis holograms from the DHM QPI measurements were reconstructed by a variant of the Fourier transformation method as described before [37] and, if required, numerically refocused by using a convolution approach [36]. The obtained QPI images for every position and time point were averaged to reduce coherence-induced image disturbances [38] and subsequently processed by the ImageJ (version 2.14.0/1.54f) feature “Subtract background” based on the rolling ball algorithm [39] to compensate for irregular spatial phase background fluctuations. From the QPI images, the dry mass (*dm*) [40] of the whole cell population within the observed FOV (*S*_FOV_ = 405 µm × 538 µm) was calculated at the beginning of the measurement (t = 0) and after 24 h (t = 24), as described in Equation (1). The dry mass was values were obtained from the cell-induced mean phase shift $\Delta \bar{\varphi }$ [41], using ImageJ version 2.14.0/1.54f via custom-built scripts, as described previously [28], from the light wavelength of the utilized laser (λ = 532 nm) and from the specific refractive index increment, which relates the phase shift to the intracellular protein content (*α* = 0.19 × 10^−3^ mm^3^/g) [42].

(1) $$dm=\frac{\lambda }{2\pi \alpha }\Delta \bar{\varphi }S_{FOV}$$

The dry mass values were used to quantify the dry mass increment (*DMI*) of the cell populations at each position in relation to the values at t = 0, respectively (Equation (2)), which was subsequently normalized to the maximal dry mass value.

(2) $$DMI={dm}_{t=24}-{dm}_{t=0}$$

### 2.6. WST-8 Cell Viability Assay

The remaining cell populations in the 96-well plate were washed with 200 µL of fresh cell culture medium. A total of 200 µL of WST-8 working medium (cell culture medium, 0.7 mM WST-8, 1-m PMS 0.04 mM) was added to the cells and incubated for 60 min at 37 °C and with 5% CO_2_. Afterwards, the light absorption of the reduced WST8 was detected with a spectrophotometer (CLARIOstar, BMG Labtech, Ortenberg, Germany) at 450 nm. For light wavelengths < 600 nm, absorbance measurements can be affected by cell- and particle-induced light scattering effects. Therefore, reference measurements of the WST-8 and LDH assays were performed in a commonly utilized wavelength range at 620 nm. Four technical repeats were measured for each nanoparticle concentration and control (*n* = 4) (Figure 1D).

The evaluation of cellular metabolic activity was performed as described in detail before [43]. Therefore, each measured OD_450nm_ value of the wells with cell populations was corrected with the mean values of the background controls (wells without cells) to calculate out the spontaneous reactivity of the cell culture medium and WST-8. The OD_620nm_ was subtracted to calculate out any possible turbidity caused by cells or nanoparticles. Afterwards, the relative cell viability was calculated in comparison to the resulting value of the negative cell culture medium control, which was set to 100% (Equation (3)).

(3) $$Cell\;Viability\left(\%\right)=\frac{{OD}_{450-620,\;sample}-{OD}_{450-620,\;background}}{{OD}_{450-620,\;neg.control}-{OD}_{450-620,\;background}}\times 100$$

### 2.7. LDH Release Cell Death Assay

In each well of the 96-well plate with the supernatant of the cell populations, 100 µL of INT working solution (lactic acid 56 mM, PMS 0.28 mM, INT 0.66 mM, NAD 1.3 mM) was added, and the light absorption at 492 nm was immediately measured by a spectrophotometer (CLARIOstar, BMG Labtech, Ortenberg, Germany). To obtain kinetic data of the LDH activity, all the wells were measured every minute for 30 min (Figure 1E).

To determine the release of the LDH enzyme, which increases proportionally with cell lysis, OD_492 nm_ values of the LDH reaction were plotted for each time point, and for the linear range, a regression slope was fitted. The mean OD_492 nm_ of the cell culture medium control was subtracted from the mean OD_492 nm_ of the samples to correct for the amount of LDH in the supernatant of viable cells. The relative toxicity in comparison to the cytotoxicity-positive control digitonin was calculated as described previously [43] (Equation (4)), which was rated as 100% *LDH release* (100% cell death) and was applied as a 100% scale basis for all the other mean values and standard deviations.

(4) $$LDH\;release\left(\%\right)=\frac{m_{OD492,\;sample}-m_{OD492,\;neg.control}}{m_{OD492,\;pos.control}-m_{OD492,\;neg.control}}\times 100$$

### 2.8. Calculation of EC_50_ Values and Statistical Analysis

Data were obtained from three independently performed experiments (*n* = 3) and evaluated with the software Graphpad Prism version 8.4.3. Mean EC_50_ values [33,34] with standard deviations were calculated by dose–response curves, which were determined by plotting the relative dry mass increment values determined by DHM, the relative cell viability data obtained by WST-8 and the relative cytotoxicity values determined with LDH for each nanoparticle concentration, measurement and fitting of a nonlinear regression with four parameters to the data points. R^2^ values were used to evaluate the fit and resulting EC_50_ values (Figure 1F). ANOVA was performed for the statistical analysis of the assay results, and significance levels were given as *p* < 0.001 (***), *p* < 0.01 (**) and *p* < 0.05 (*).

## 3. Results

### 3.1. Qualitative Analysis of Cell Proliferation and Morphology Alterations in DHM QPI Images after Incubation with PACA Nanoparticles

**Figure 2:**
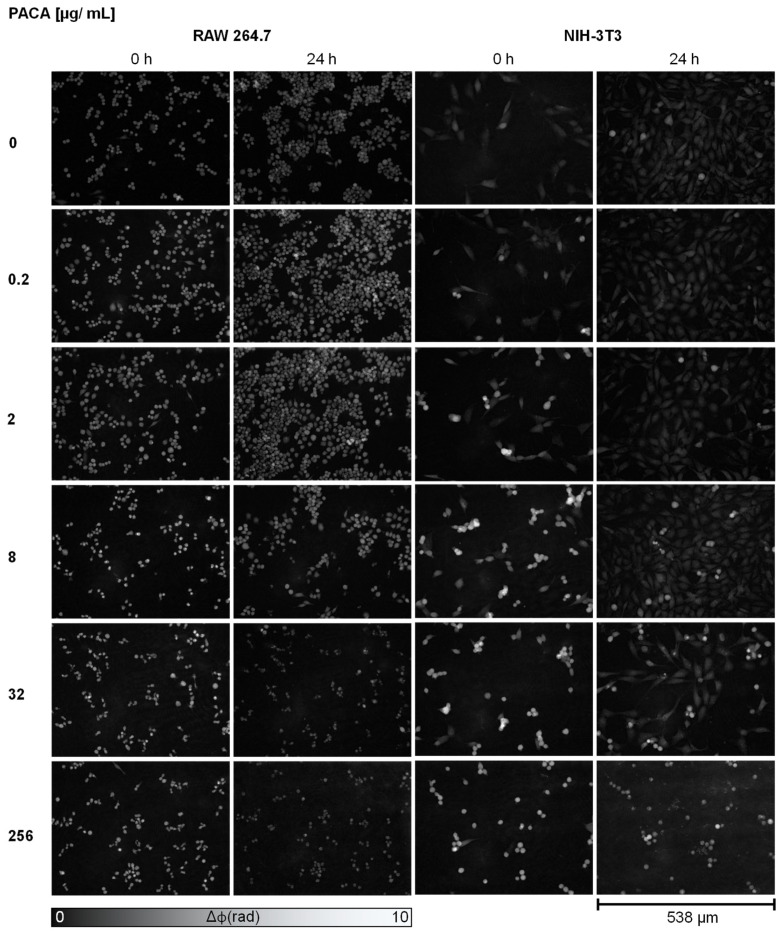
DHM QPI images of RAW 264.7 macrophages and NIH-3T3 fibroblasts incubated with unloaded PACA nanoparticles in five representatively selected concentrations (0.2, 2, 8, 32 and 256 µg/mL) vs. cell culture medium controls (0 µg/mL) at time points t = 0 and t = 24 h. For both cell lines, viable proliferated cells were observed after incubation with cell culture medium control and 0.2 and 2 µg/mL of unloaded PACA nanoparticles. RAW 264.7 cells with 8 µg/mL showed cell debris at t = 0, and after 24 h; NIH-3T3 cells showed cell detachment at t = 0 and proliferated cells after 24 h. For 32 and 256 µg/mL of unloaded PACA nanoparticles, cell debris was observed for RAW 264.7 macrophages after 24 h, and proliferated cells, detached cells and cell debris were observed for NIH-3T3 with 32 µg/mL. Corresponding bright-field images (Figure S1) and enlarged areas of DHM QPI and bright-field images (Figure S2), which allow for a more detailed investigation of the cellular morphology alterations, are provided in the Supplementary Materials.

**Figure 3:**
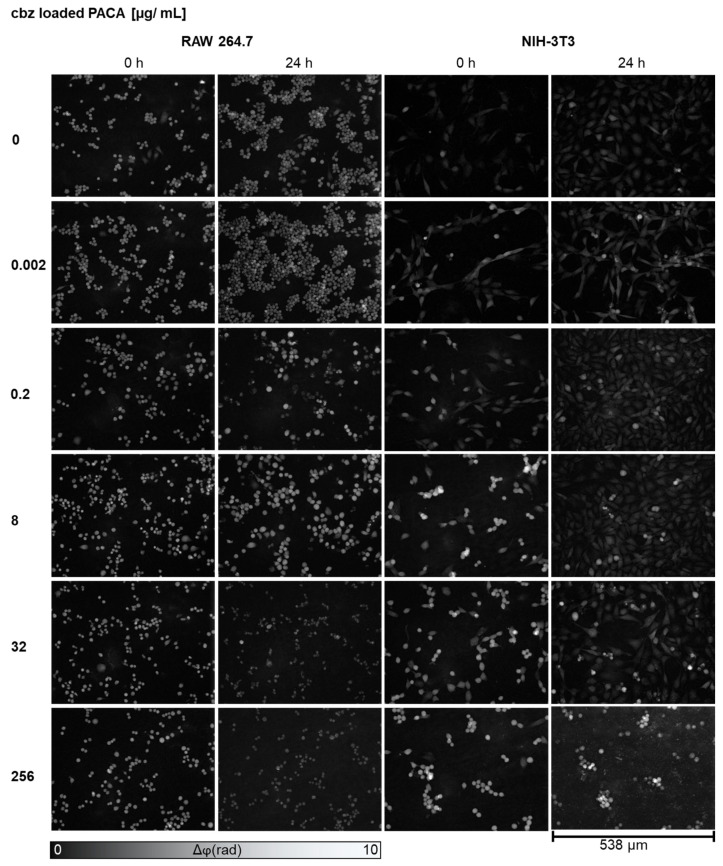
DHM QPI images of RAW 264.7 macrophages and NIH-3T3 fibroblasts after incubation with cbz-loaded PACA nanoparticles in five representatively selected concentrations (0.002, 0.2, 8, 32 and 256 µg/mL) vs. cell culture medium controls (0 µg/mL) at time points t = 0 and t = 24 h. For both cell lines, after incubation with cell culture medium control and 0.002 µg/mL of cbz-loaded PACA nanoparticles, viable cells were detected after 24 h. For 0.2 µg/mL, cell debris could be observed for RAW 264.7, and detached and swollen cells could be observed for NIH-3T3. Macrophages incubated with 8 µg/mL of cbz-loaded PACA showed a swollen but viable cell morphology after 24 h, and for NIH-3T3, detached cells and cell debris were visible at t = 0 24 h, but after 24 h, proliferated cells were visible. Cell debris was observed in both cell lines with 32 and 256 µg/mL of cbz-loaded nanoparticles after 24 h. Corresponding bright-field images (Figure S3) and enlarged areas of DHM QPI and bright-field images (Figure S4), which allow for a more detailed investigation of the cellular morphology alterations, are provided in the Supplementary Materials.

Initially, the DHM QPI images of RAW 264.7 and NIH-3T3 cells were qualitatively evaluated for cell proliferation and changes in cell morphology. Information on the cytotoxicity of particles can be obtained by evaluating cell proliferation and morphological aspects such as cell rounding. As the reasons for rounded cells in a cell population vary and include dying as well as dividing or migrating cells, only a significantly increased frequency of rounded cell morphologies can be attributed to cell death. Five concentrations were analyzed vs. controls for each nanoparticle type at the time points t = 0 and t = 24 h (Figure 2 and Figure 3). Figure 2 shows representative images of the cells after incubation with unloaded PACA nanoparticles and controls. Corresponding bright-field images of Figure 2 (Figure S1) are provided in the Supplementary Materials. Enlarged areas of the DHM QPI and bright-field images of Figure 2, which allow for a more detailed investigation of the cellular morphology alterations, are provided in the Supplementary Materials (Figure S2). Within the 24 h observation time frame, for both the RAW 264.7 macrophages and NIH-3T3 fibroblasts, proliferating viable cells were observed in the culture medium control (0 µg/mL). After incubation with 0.2 and 2 µg/mL of unloaded PACA nanoparticles, at t = 24 h, for both cell lines, viable cells and a similar proliferation as in the medium control were evident. At 8 µg/mL of unloaded PACA nanoparticles, for the RAW 264.7 and NIH-3T3 cells, cell debris and cell detachment were already apparent at time point t = 0 (see orange-colored arrows in Figure S2); however, after 24 h, viable and proliferated NIH-3T3 cells were detected (see blue-colored arrows in Figure S2). The cells incubated with unloaded PACA nanoparticle concentrations of 32 and 256 µg/mL showed cell debris in both cell lines at t = 0 h and after 24 h (see green-colored arrows in Figure S2). Except for the NIH-3T3 fibroblasts with 32 µg/mL, which proliferated in a reduced manner compared to the medium control cells and detached cells could be detected after 24 h (see yellow-colored arrows in Figure S2).

Figure 3 presents DHM QPI images of the RAW 264.7 and NIH-3T3 cells after incubation with cbz-loaded PACA nanoparticles and the control. Corresponding bright-field images of Figure 3 (Figure S3) are provided in the Supplementary Materials. Enlarged areas of the DHM QPI and bright-field images, which allow for a more detailed investigation of the cellular morphology alterations, are provided in the Supplementary Materials (Figure S4). For both cell lines, incubation with 0.002 µg/mL of cbz-loaded PACA nanoparticles did not cause any obvious morphology alterations, and after 24 h, viable and proliferating cells, similar to the medium control cells, were detected. For 0.2 µg/mL of cbz-loaded PACA nanoparticles, cell debris was observed for the RAW 264.7 macrophages after 24 h (see orange-colored arrows in Figure S4). At 8 µg/mL of cbz-loaded PACA, after 24 h, the macrophages showed a swollen but viable cell morphology (see blue colored arrows in Figure S4). In contrast, at this nanoparticle concentration, the NIH-3T3 cells showed a detached morphology at t = 0 but proliferated cells after 24 h (see green colored arrows in Figure S4). At 32 and 256 µg/mL of cbz-loaded nanoparticles, cell debris was observed in both cell lines after 24 h (see yellow-colored arrows in Figure S4).

In summary, the DHM QPI images allowed for an initial qualitative analysis of the changes in cell morphology and proliferation, providing first insights into the nanoparticle cytotoxicity and cellular responses prior to quantitative endpoint determinations with DHM, WST-8 and LDH. The first effects of the unloaded PACA particles on the RAW 264.7 macrophages and NIH-3T3 fibroblasts could be observed at 8 µg/mL, while those loaded with cbz already had effects on cell morphology and proliferation at a lower concentration of 0.2 µg/mL.

### 3.2. Multimodal Cytotoxicity Assessment by DHM with Downstream WST-8 and LDH

**Figure 4:**
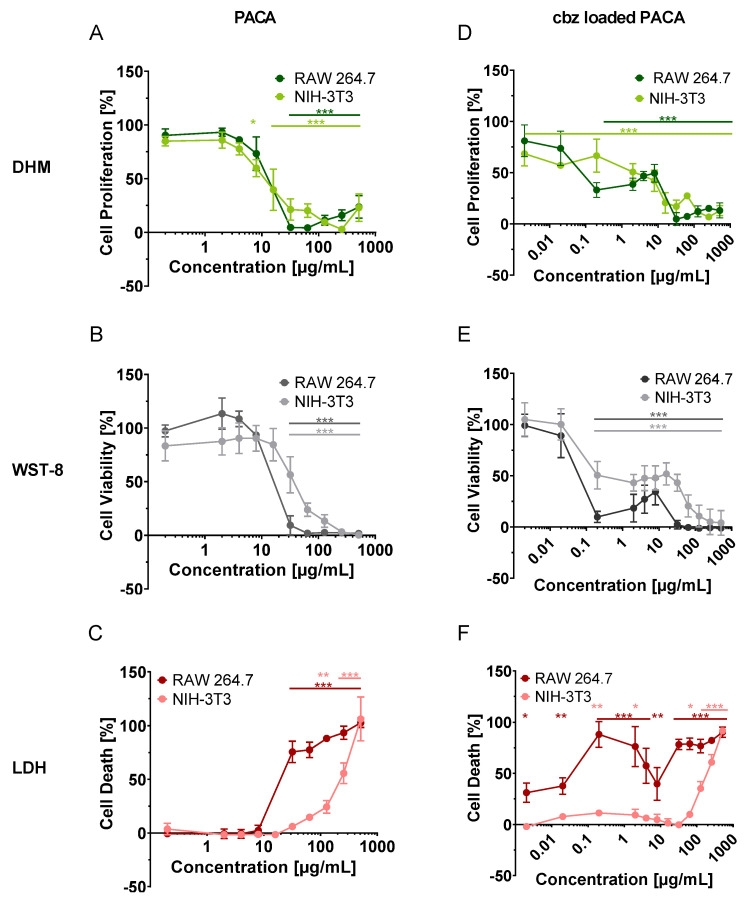
Dose–response relationship for unloaded PACA and cbz-loaded PACA nanoparticles on cell proliferation (DHM, green, (**A**) unloaded (**D**) cbz-loaded PACA), viability (WST-8, gray, (**B**) unloaded (**E**) cbz loaded PACA) and death (LDH, red, (**C**) unloaded (**F**) cbz loaded PACA) of RAW 264.7 macrophages and NIH-3T3 fibroblasts. Mouse RAW 264.7 macrophages and NIH-3T3 fibroblasts were seeded in 96-well plates incubated with unloaded and cbz-loaded PACA, and dry mass increments of cell populations were analyzed with DHM. Subsequently, the viability of the same cell populations was determined with a WST-8 metabolic activity assay, and the supernatants were analyzed in parallel for the release of LDH to detect cell death. The mean values ± SD from three independent experiments are shown (*n* = 3). Significance levels were given as *p* < 0.001 (***), *p* < 0.01 (**) and *p* < 0.05 (*).

Figure 4 shows the results from the multimodal cytotoxicity assessment approach with DHM and downstream WST-8 and LDH. Therefore, the dose–response relationships of unloaded and cbz-loaded PACA nanoparticles on the RAW 264.7 macrophages and NIH-3T3 fibroblasts were determined for the relative cell proliferation retrieved by DHM (Figure 4A,D), cell viability detected by the WST-8 assay (Figure 4B,E) and cell death determined by the LDH assay (Figure 4C,F). EC_50_ values were obtained by fitting the dose–response curves to the averaged data of *n* = 3 experiments, and R^2^ values were considered to assess the reliability of the curves, as described in Section 2.6 “Calculation of EC_50_ values and statistical analysis”.

For the RAW 264.7 cells treated with unloaded PACA nanoparticles, no effect on the relative cell proliferation was observed in the DHM assay for 0.2, 2, 4 and 8 µg/mL compared to the cell proliferation of the medium control (Figure 4A). Similarly, no effect on the relative cell viability, determined by the WST-8 assay, as well as on relative cell death as a result of the LDH assay could be detected for these concentrations (Figure 4B,D). A highly significantly (*** *p* < 0.001) reduced relative cell proliferation and viability and relative increased cell death could be observed with all assay types, with the unloaded PACA NP concentrations ranging from 32 to 512 µg/mL (Figure 4A–C). Furthermore, a moderately increasing relative cell proliferation (DHM) was observed again for 128–512 µg/mL of unloaded PACA nanoparticles (Figure 4A). However, this effect was not observed with the WST-8 and LDH assays. Dose–response curves (R^2^ DHM: 0.92 ± 0.03 (Figure 4A), WST-8: 0.96 ± 0.01 (Figure 4B), LDH: 0.98 ± 0.01 (Figure 4C)) with EC_50_ values of between 9 and 22 µg/mL could be determined (DHM: 9 ± 1 µg/mL (Table 1), WST-8: 14 ± 1 µg/mL (Table 1), LDH: 22 ± 4 µg/mL (Table 1)). The EC_50_ values from the cell proliferation retrieved by DHM were lower in comparison to those from LDH and WST-8.

Similarly to the macrophages, the NIH-3T3 fibroblasts show no altered relative cell proliferation (DHM, Figure 4A), viability (WST-8 Figure 4B) or cell death (LDH, Figure 4C) for concentrations of 0.2, 2 and 4 µg/mL of unloaded PACA nanoparticles. Significant effects (* *p* < 0.05) on the relative cell proliferation could be determined for 8 µg/mL (DHM, Figure 4A). In contrast, the calculated relative cell viability was not affected up to 32 µg/mL of unloaded PACA nanoparticles (WST-8, Figure 4B), nor was the relative cell death affected up to 64 µg/mL (LDH, Figure 4C). The maximum effect on cell proliferation (DHM) and on cell viability (WST-8) was reached for nanoparticle concentrations >64 µg/mL. Significant effects (** *p* < 0.01) on the relative cell death were detected starting with 128 µg/mL (*** *p* < 0.001 for 256 and 512 µg/mL), and the maximum effect was reached at higher concentrations with LDH than with the DHM and WST-8 assays (LDH, Figure 4C). Dose–response curves (R^2^ DHM: 0.86 ± 0.06 (Figure 4A), WST-8: 0.94 ± 0.01 (Figure 4B), LDH: 0.97 ± 0.01 (Figure 4C)) with EC_50_ values were calculated for DHM, with 15± 8 µg/mL (Table 1), and WST-8, with 40 ± 4 µg/mL (Table 1). For the LDH assay, the calculated EC_50_ value was higher, with 311 ± 116 µg/mL (Table 1).

For both cell lines treated with cbz-loaded PACA, no monotonic dose–response curve could be fitted with all the assay types (Figure 4D,E), except for the NIH-3T3 cells in the LDH assay (Figure 4F). Therefore, no EC_50_ values could be determined for this particle type. The relative cell proliferation (DHM, Figure 1D) and cell viability (WST Figure 1E) of the RAW 264.7 cells were not affected with 0.002 and 0.02 µg/mL of cbz-loaded PACA. However, the relative cell death was significantly increased for 0.002 (* *p* < 0.05) and 0.02 µg/mL (** *p* < 0.01) of cbz-loaded PACA (LDH, Figure 4F) compared to the medium control cells. The relative cell proliferation, viability and death of the RAW 264.7 cells were highly significantly (*** *p* < 0.001) affected by concentrations starting at 0.2 µg/mL (Figure 4D–F). A re-increasing relative cell proliferation and cell viability and a decreasing cell death at 4 and 8 µg/mL could be observed in the results of all the assay types (Figure 4D–F), which nevertheless showed highly significant differences to the medium control (*** *p* < 0.001). Even higher concentrations of between 32 and 512 µg/mL again led to the maximum effect on the relative cell proliferation, viability and cell death of the RAW 264.7 macrophages (*** *p* < 0.001).

For the NIH-3T3 fibroblasts, highly significant (*** *p* < 0.001) differences in terms of the relative cell proliferation compared to the medium control could be detected with all the tested concentrations of cbz-loaded PACA (DHM, Figure 4D). The results of the WST-8 and LDH biochemical assays showed no effects up to 0.2 µg/mL (Figure 4E,F). While highly significant (*** *p* < 0.001) differences in relative cell viability could be detected in WST-8, starting at 0.2 µg/mL, compared to the medium control, only significant differences (* *p* < 0.05, ** *p* < 0.01) could be detected with the LDH assay, and a similar effect to that in the toxicity control, digitonin (100%), could only be achieved with 512 µg/mL of cbz-loaded PACA particles (*** *p* < 0.001, Figure 4F). For the fibroblasts, the same trend was observed as for the macrophages. With 4 and 8 µg/mL of cbz-loaded PACA, a renewed increase in cell proliferation and viability was observed, which decreased again with even higher concentrations (Figure 4D,E). Therefore, the numerical fitting of a monotonic dose–response curve was not possible. For LDH, however, this observation could not be made for the NIH-3T3 cells (Figure 4F).

In summary, the dry mass increase obtained by DHM was more sensitive for the detection of the cytotoxicity of unloaded PACA nanoparticles than the cell viability determined by WST-8 and the measurement of cell death with LDH. The effects of the particles loaded with cbz were observed at lower concentrations than those of the unloaded particles. Furthermore, due to a non-monotonic dose–effect relationship, no EC_50_ values could be generated to compare the three methods by one parameter, but the experimental data showed a similar trend.

## 4. Discussion

In our study, a label-free digital holographic microscopy QPI cell proliferation assay was evaluated in comparison to the commonly used biochemical assays WST-8 and LDH to assess the cytotoxic potential of unloaded and cbz-loaded PACA nanoparticles. The compatibility and performance of a marker-free QPI test system was analyzed in comparison to common colorimetric in vitro assay methods, which included several staining and washing steps. A multimodal experimental design was used, in which the same cell populations of RAW 264.7 macrophages and NIH-3T3 fibroblasts were measured in the same well plate with each method, and EC_50_ values were determined for comparability.

A dose-dependent effect of the unloaded PACA nanoparticles on the two cell lines could be observed in all the experiments, i.e., in the relative cell proliferation quantified by DHM (Figure 4A), in the relative cell viability retrieved by the WST-8 assay (Figure 4B) and in the relative cell death measured by the LDH assay (Figure 4C). These relationships allowed for the determination of the mean EC_50_ values via a sigmoidal dose–response curve.

The particles loaded with the cytostatic drug showed higher toxic effects than the unloaded particles (Figure 4A–C). However, in contrast to the unloaded particles (Figure 4A–C), no monotonic dose-dependent toxicity was observed. With all three assays, DHM as well as WST-8 and LDH, a recurring increase in proliferation and viability in combination with an accompanied decrease in cell death could be observed at concentrations of 4 and 8 µg/mL (Figure 4D–F). This effect prevented the calculation of accurate EC_50_ values based on a sigmoidal dose–response curve and, thus, prevented a direct comparison of the methods. It is striking that no EC_50_ value could be determined with any of the applied methods for the cbz-loaded PACA nanoparticles. Nevertheless, the toxicity assessments of the particles based on the results of the individual methods led to very similar results (Figure 4D–F). A deeper analysis of the non-monotonic dose–effect relationship of the cbz-loaded PACA particles used in this study and from the literature is presented in the Supplementary Materials (Information S1).

Due to the difficulty in obtaining the EC_50_ values for the cbz-loaded PACA nanoparticles, it is more challenging to compare the DHM QPI results with the results of the WST-8 and LDH assays and to determine its capabilities and sensitivity for cytotoxicity assessment. A qualitative analysis of the DHM QPI images provided further indications of a non-monotonic dose–effect relationship between the particles loaded with cbz. Three-dimensional surface plots of the QPI images with superimposed bright-field image textures were created to provide a more detailed representation of these effects (Figure S5). The RAW 264.7 macrophages treated with 0.2 µg/mL of cbz-loaded PACA showed more cell debris than the medium control cells and cells incubated with an 8 µg/mL concentration of the particles, which indicated more viable cells (see black arrows in Figure S5). Consequently, it can be summarized that if EC_50_ values cannot be obtained, DHM can provide further information on effects that are difficult to quantify, in contrast to WST-8 and LDH.

The determined EC_50_ values in our results showed a cell-line-dependent toxicity of the unloaded PACA particles (Figure 4A–F), with a higher sensitivity for the RAW 264.7 macrophages compared to the NIH-3T3 fibroblasts. A detected higher sensitivity of macrophages to PACA particles was also observed in earlier studies by Eder et al. [28,43], and other studies have described a cell-line-dependent cytotoxicity of nanoparticles as well [35,44,45,46]. A possible reason could be that the interactions and uptake of nanomaterials can vary for different cell types [47,48]. Macrophages are phagocytotic cells, and they thus show a different uptake behavior to endocytotic cells such as NIH-3T3 fibroblasts, resulting in a higher particle load and different toxicity mechanisms. [30]

In addition, our results showed a method-dependent variation, as indicated by the different EC_50_ values for each assay type. Variations in assay results have already been described in the literature [49], explaining that the varying sensitivity of the assays makes it difficult to obtain consistent dose–response data for different toxicity endpoints and different readout systems [8]. The EC_50_ values in our study with the different endpoints for the unloaded PACA particles with the macrophages were very close to each other (Figure 4A–C). The values determined with DHM were the lowest (Figure 4A) for both the RAW 264.7 macrophages and the NIH-3T3 fibroblasts and were closer to the WST-8 results (Figure 4B) than those of the LDH assay, which provided the highest values (Figure 4C). This can be explained by the fact that the DHM and WST-8 assays determined more similar endpoints compared to the LDH assay. While DHM measures cell proliferation and WST-8 measures cellular metabolic activity, both being indicators of cell viability, the LDH assay detects membrane damage as an indicator for cell death and, thus, it provides a different endpoint. The different endpoints could explain the different EC_50_ values obtained with DHM and LDH on the same cell populations. The lower sensitivity of the LDH test in our study compared with DHM (Figure 4) can be explained by the membrane damage and the leakage of the lactate dehydrogenase enzyme from the cells, which are analyzed as cytotoxic effects in LDH assays. The effects of particles that influence intracellular activities but do not initially have any influence on membrane integrity are not detected by LDH release assays [49]. It has already been reviewed in the literature that the LDH test has a lower sensitivity than other cytotoxicity assays [50]. A lower sensitivity of LDH assays for testing PACA particles in particular is described in a study by Sulheim et al., in which the effects of PACA particles on pig kidney cells (LLC-PK1) and human liver cells (Hep G2) were observed in an LDH assay at higher concentrations than in an MTT assay [30]. The sensitivity and results of an assay are dependent on optimal conditions. To consider these in experimental design, it is important to know the critical factors such as the tested cells and the densities and dosage of the investigated substances as well as their incubation time [50]. This relevance is reported in a study on human HL-60 promyeloblastic and HepG2 liver cells by Riss et al., where both the number of seeded cells and the cell density of the parent stock culture had an influence on the toxic effects of tamoxifen and vinblastine, as measured by ATP and LDH assays. Furthermore, they showed that the maximum LDH release in the LDH assay was lower after 24 h of incubation with tamoxifen than after shorter incubation times, which was likely because of inactivation by proteases released from the dying cells [51]. These results may provide a further explanation for the lower sensitivity of the LDH assay in our study. Nevertheless, the integration of LDH assays into the workflow for testing PACA particles ensures a better understanding of the different aspects of cell function and integrity and the biological activity of PACA nanoparticles, as these degradation products can cause a perforation of the cell membrane [52]. Despite this, the results demonstrate the importance of optimizing the conditions of cytotoxicity assays in order to obtain the most accurate and sensitive results, and they show that the time frame should not be extended to 48 h in order to prevent the LDH from being completely degraded by proteases. At the same time, the results show the challenge of utilizing a multimodal approach when quantifying the toxicity of nanomaterials. In order to achieve a fast and comprehensive screening of particles in one workflow, a compromise for cell densities and incubation times for all assays must be defined and considered.

A further challenge in colorimetric assays for determining the effects of nanoparticles was mentioned before, as particles can interfere with the assay components, which may result in misleading results [8]. In contrast, DHM is expected to have minimal interactions with the biological sample and the nanoparticles due to its robustness against moderate absorption effects and incoherent fluorescent light. A potential influence on the results of DHM measurement may be the changes in the cellular dry mass that are induced by the incorporated particles. However, in our study with DHM-based QPI, we could not detect such any dry mass alterations within the application-relevant particle concentration ranges and the measurement uncertainty, but at higher particle doses (256 and 512 µg/mL), a slight dry mass increment increase was observed. A possible explanation of this effect could be that the high particle concentrations led to an effect on the optical quality of the QPI images due to light scattering effects, which resulted in a higher background noise level and, consecutively, in a lower signal-to-noise ratio. The increased background noise level could have led to overestimated dry mass values for the higher particle concentrations, as indicated in Figure 4A,D by the slightly increased cell proliferation results for the concentrations of 128, 256 and 512 µg/mL. This optical effect illustrates the limitations of DHM-based assays at high particle concentrations. However, the measurements of the unloaded PACA particles with DHM QPI without cell populations showed an increase in the mean phase contrast for the concentrations of 256 and 512 µg/mL (Figure S6). An increased background noise level can lead to overestimated dry mass values and thus has to be considered when measuring high particle concentrations with DHM QPI, and measurements of the image background should be performed to achieve high-quality and reliable results. For future prospects, the dry mass values of the cell populations could be corrected with the dry mass values of the background to obtain only the data of the cell populations in the FOVs.

Biochemical methods in cytotoxicity assessment provide information about the presence or absence of a functional mechanism in cells. WST-8 assays, for example, can provide information on cell viability if there is an effect on metabolic activity, and LDH assays can mainly provide information if the cell membrane loses its integrity due to necrosis. This effect is analyzed at a specific endpoint, which is determined before the experiment, thus averaging the effect of the whole cell population. Complex temporal interactions as well as individual cell responses are not considered, and one specific mechanism can often be determined by each assay type. Microscopy-based screenings, and DHM QPI in particular, can facilitate proliferation measurements of cell populations and can detecting changes in the migration and phenotype of individual cells down to the nano scale over a period of time [23], which could provide detailed information about biological processes [23,53]. The advantages of microscopy-based methods for the determination of drug effects on cells are described in the literature. For example, a study using automated fluorescence microscopy showed that the lowest effective concentration of a drug and its specificity can be efficiently determined based on the cellular phenotype [54]. Changes in cell morphology that were specifically induced by an effect of nanoparticles on cell viability and that were quantified with QPI are presented in a study by Lai et al. [55]. Here, the effects of gold nanorods on murine macrophages (J774A.1) were measured with a WST-8 assay, and the changes in morphology were measured with DHM QPI in the form of the dry mass and cell area. The results showed that, although qualitatively, no changes in cell morphology were visible at first sight, they could be quantified with DHM QPI [55].

Technological advances can further improve the detection of subpopulations and phenotypes for the toxicity screening of nanoparticles using microscopy-based methods. Sophisticated image evaluation algorithms that, e.g., rely on convolutional neural networks (CNNs) [56] can potentially provide improved biophysical parameter extraction and are suitable for subsequent advanced analysis with machine learning approaches [57,58]. However, these advantages of microscopy-based methods are also linked to some challenges. Image acquisition is crucial for microscopy-based methods, as it determines the image quality and thus the overall screening results quality. In our study, the DHM image quality was related to how many images were acquired with the ETL to reduce coherence-induced image disturbances [38]. As measurements from a 96-well plate can rapidly result in up to several terabytes of data in one experiment, this also means that there are challenges in terms of handling and storage [53]. In our study, we used custom-built software for the DHM measurements. The use of software can streamline the process in a user-friendly manner and reduce limitations concerning the acquisition time (typically to 20–30 min), and it can be operated by individuals with basic assay and light microscopy knowledge. The rapid image acquisition (in milliseconds) allows for measurements in non-vibration isolated environments. In our study, 192 FOVs from a 96-well plate were imaged within a 30 min interval. The assay-specific developed software enabled automated QPI image reconstruction, and optimization at individual positions was only required for exceptional events like defocusing effects or light scattering caused by serum particles or cell debris. Moreover, the data management features of the custom-built DHM assay software enabled the managing of image stacks and metadata, which allowed for an accelerated automated data evaluation of the QPI images for dry mass determination using the freely available software ImageJ via custom-built scripts. This reduced the duration of the entire evaluation process in our study, including reducing the time required for the QPI image reconstruction and dry mass calculation for the measurements with two time points (t = 0 h, t = 24 h) in the 96-well plate to a few minutes.

In summary, the different sensitivities of DHM, WST-8 and LDH assays due to the different measured endpoints and the potential for particles to interact with the biochemical test systems demonstrate that a matrix of different assays is required to assess the toxicity of nanomaterials. Furthermore, these results show that a multimodal assay provides complementary information about nanoparticle-induced cellular effects. Finally, the results demonstrate that cell proliferation measurement by DHM is a suitable tool for the cytotoxicity screening of nanoparticles, as it can detect early effects of unloaded PACA particles on cell lines and thus offers a good alternative to biochemical assays with which nanoparticles may interfere. In the context of research in the emerging field of nanomaterials for medical applications [1,2], particularly for imaging applications in tumor diagnostics or for drug delivery for cancer treatment, DHM can be a valuable alternative cytotoxicity assessment method, and it has a special benefit for testing new nanoparticles from different materials due to its minimal interactions with various particle properties. Moreover, DHM can also be used broadly for assessing engineered nanoparticles and the environmental impacts of nanoparticles within the life cycle assessment of nanoparticles.

## 5. Conclusions and Future Perspectives

A multimodal approach was verified in this study, in which the effects of unloaded and cabazitaxel-loaded poly(alkyl cyanoacrylate) nanoparticles were investigated using DHM and two downstream biochemical methods. The dry mass increase in the cell populations was determined over 24 h using DHM, which was followed directly by an investigation of their cell viability using a WST-8 assay and the detection of their cell death by analyzing the release of the enzyme lactate dehydrogenase (LDH) in the supernatant of the cell populations. By determining the half-maximal effective concentration (EC_50_), the sensitivity and performance of DHM was directly compared to the other two methods. Our results highlight QPI-based DHM as a bioimaging method to monitor and quantify the effects of polymeric nanoparticles on cells, as it has potential due to its minimally invasive characteristics that can minimize interferences between the particles and assay components, which may occur with biochemical assays used in toxicity screening. In addition, the results show that DHM can be efficiently integrated into experimental routines and existing workflows for cytotoxicity assessments in a time-efficient manner such that further results can be obtained for a comprehensive toxicity assessment of particles while limiting the overall workload. Additionally, our results show that the bioimaging method DHM provides a higher sensitivity compared to the biochemical assays WST-8 and LDH for the assessment of the toxicity of polymeric nanoparticles on RAW 264.7 macrophages and NIH-3T3 fibroblasts. Furthermore, in order to improve the study design and the precise quantification of EC_50_ values, more concentrations should be tested in the range of the inflection point. In addition, comparative measurements on the effects of free cbz could be performed to gain further insights into the role of cbz in loaded PACA nanoparticles, and further time points could be determined to assess the time-dependent effect of the particles and evaluate the performance and sensitivities of the methods at different time points. Finally, it would be valuable to explore additional parameters, such as the slope of the dose–response curve (change in response per unit dose), to compare the described methods and to highlight the advantages and disadvantages of the methods, as they differ in terms of their endpoints and sensitivity.

## Figures and Tables

**Table 1:** EC_50_ values for PACA nanoparticles on cell proliferation (DHM), viability (WST-8) and death (LDH) of RAW 264.7 macrophages and NIH-3T3 fibroblasts.

	EC_50_ [µg/mL]		
	DHM	WST-8	LDH
RAW 264.7	9 ± 1	14 ± 1	22 ± 4
NIH-3T3	15 ± 8	40 ± 4	311 ± 116

## Data Availability

Requests for data and materials should be addressed to the corresponding authors.

## Supplementary Materials

The following supporting information can be downloaded at: https://www.mdpi.com/article/10.3390/cells13080697/s1, Figure S1: Bright-field images of RAW 264.7 macrophages and NIH-3T3 fibroblasts incubated with unloaded PACA nanoparticles; Figure S2: Enlarged areas of DHM QPI images and bright-field images of RAW 264.7 macrophages and NIH-3T3 fibroblasts incubated with unloaded PACA nanoparticles; Figure S3: Bright-field images of RAW 264.7 macrophages and NIH-3T3 fibroblasts after incubation with cbz-loaded PACA nanoparticles; Figure S4: Enlarged areas of DHM QPI images and bright-field images of RAW 264.7 macrophages and NIH-3T3 fibroblasts after incubation with cbz-loaded PACA nanoparticles, Figure S5: Pseudo-3D surface plots of representative DHM QPI image overlaid with the texture of correlatively recorded bright-field images, Figure S6: DHM QPI background noise level at different concentrations of unloaded PACA nanoparticles. Information S1: Detailed analysis of non-monotonic dose–effect relationship of cbz-loaded PACA particles in this study and in the literature [59,60,61,62,63,64,65,66,67].
