# Supplementary material for: Quantitative Phase Imaging as Sensitive Screening Method for Nanoparticle-Induced Cytotoxicity Assessment
Source: Cells. 2024 Apr 17;13(8):697. doi: 10.3390/cells13080697 (PMC11049110; doi:10.3390/cells13080697)
Supplement: Supplementary file 1 [file cells-13-00697-s001.zip › cells-2926204-supplementary.pdf]

Supplementary Materials

Quantitative Phase Imaging as Sensitive Screening Method for Nanoparticle-Induced Cytotoxicity Assessment

Anne Marzi *, Kai Moritz Eder, Álvaro Barroso, Björn Kemper † and Jürgen Schnekenburger *,†

Biomedical Technology Center, University of Muenster, Mendelstraße 17, D-48149 Muenster, Germany; kai.moritz.eder@gmail.com (K.M.E.); alvaro.barroso@uni-muenster.de (Á.B.); bkemper@uni-muenster.de (B.K.)

* Correspondence: anne.marzi@uni-muenster.de (A.M.); schnekenburger@uni-muenster.de (J.S.)

† These authors contributed equally to this work.

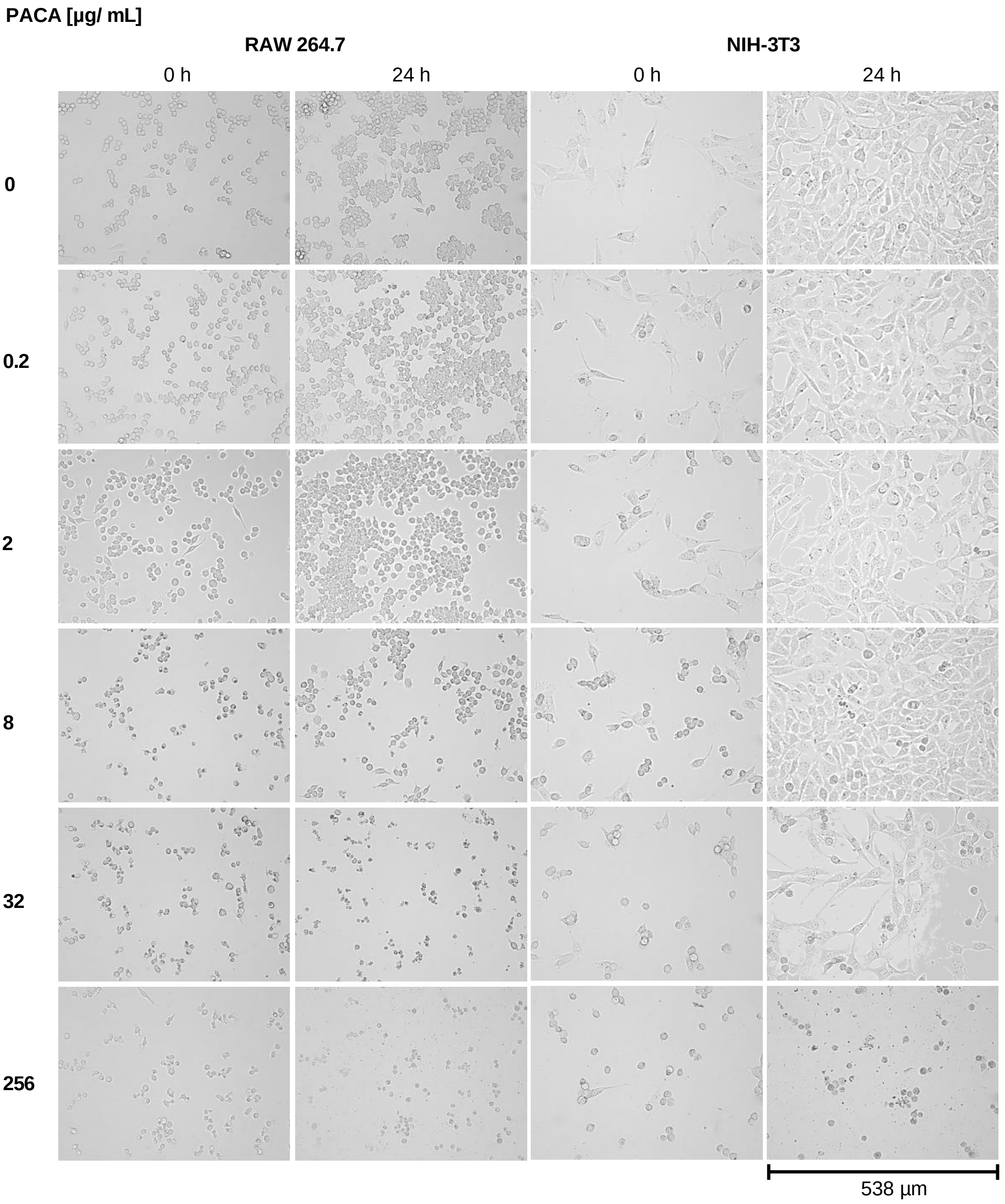

Figure S1. Bright field images of RAW 264.7 macrophages and NIH-3T3 fibroblasts incubated with unloaded PACA nanoparticles in five representatively selected concentrations (0.2, 2, 8, 32 and 256 μg/mL) vs. cell culture medium controls (0 μg/mL) at time points t = 0 and t = 24 h. For both cell lines after incubation with cell culture medium control, and 0.2 and 2 μg/mL of unloaded PACA nanoparticles viable proliferated cells are observed. RAW 264.7 cells with 8 μg/mL show cell debris at t = 0, but viable and proliferated cells after 24 hours. For 32 and 256 μg/mL of unloaded PACA nanoparticles cell debris was observed for RAW 264.7 macrophages after 24 h, detached cells and cell debris for NIH-3T3.

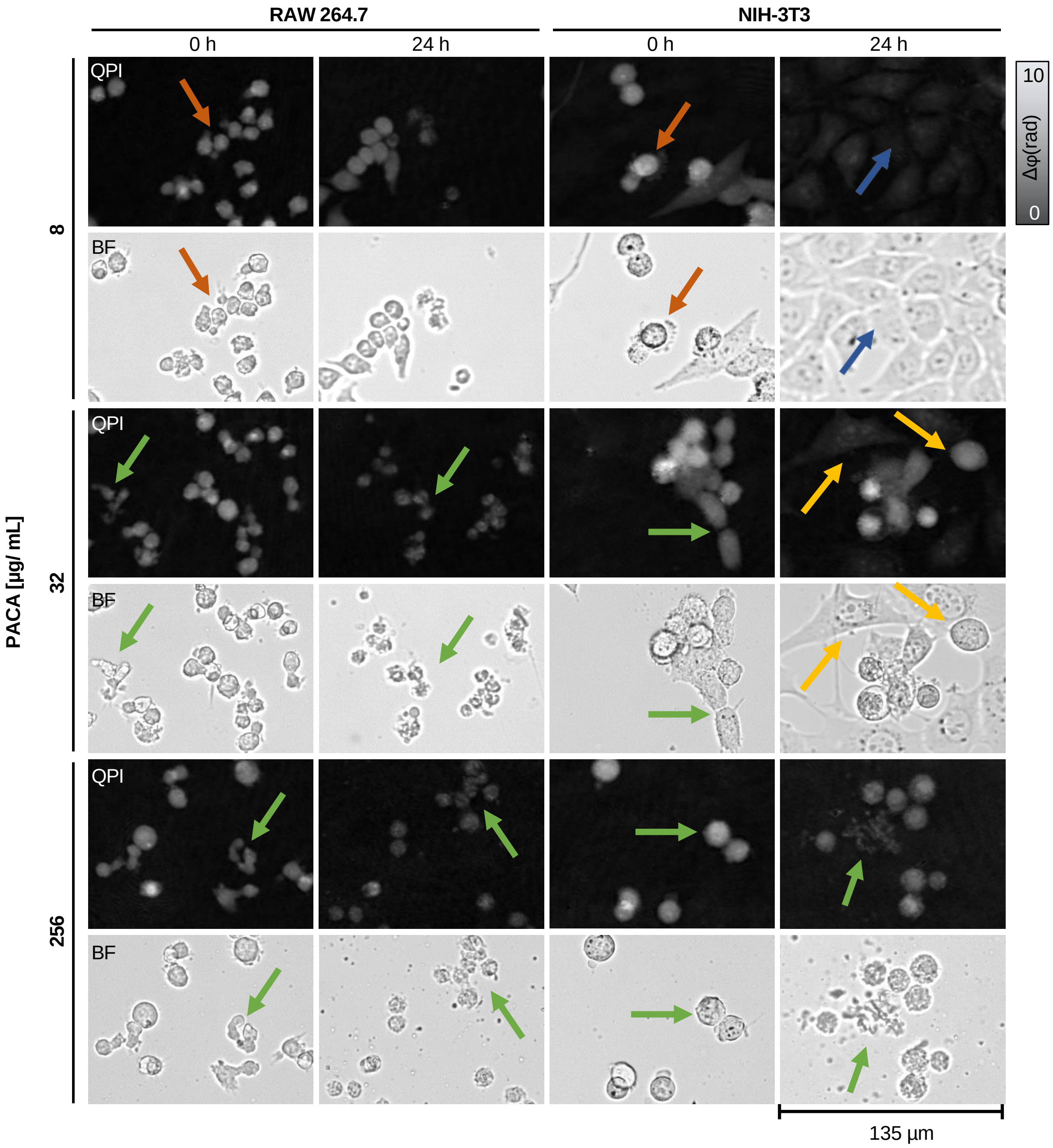

Figure S2. Enlarged areas of DHM-QPI images and bright-field images of RAW 264.7 macrophages and NIH-3T3 fibroblasts incubated with unloaded PACA nanoparticles in three representatively selected concentrations (8, 32 and 256 µg/mL) at time points t = 0 and t = 24 h. RAW 264.7 cells with 8 µg/mL show cell debris at t = 0 and after 24 hours (orange colored arrows). NIH-3T3 cells show cell detachment at t = 0 (orange colored arrows) and proliferated cells after 24 h (blue colored arrows). For 32 and 256 µg/mL of unloaded PACA nanoparticles cell debris was observed for RAW 264.7 macrophages after 24 h (green colored arrows), proliferated, detached cells and cell debris for NIH-3T3 with 32 µg/mL (yellow colored arrows).

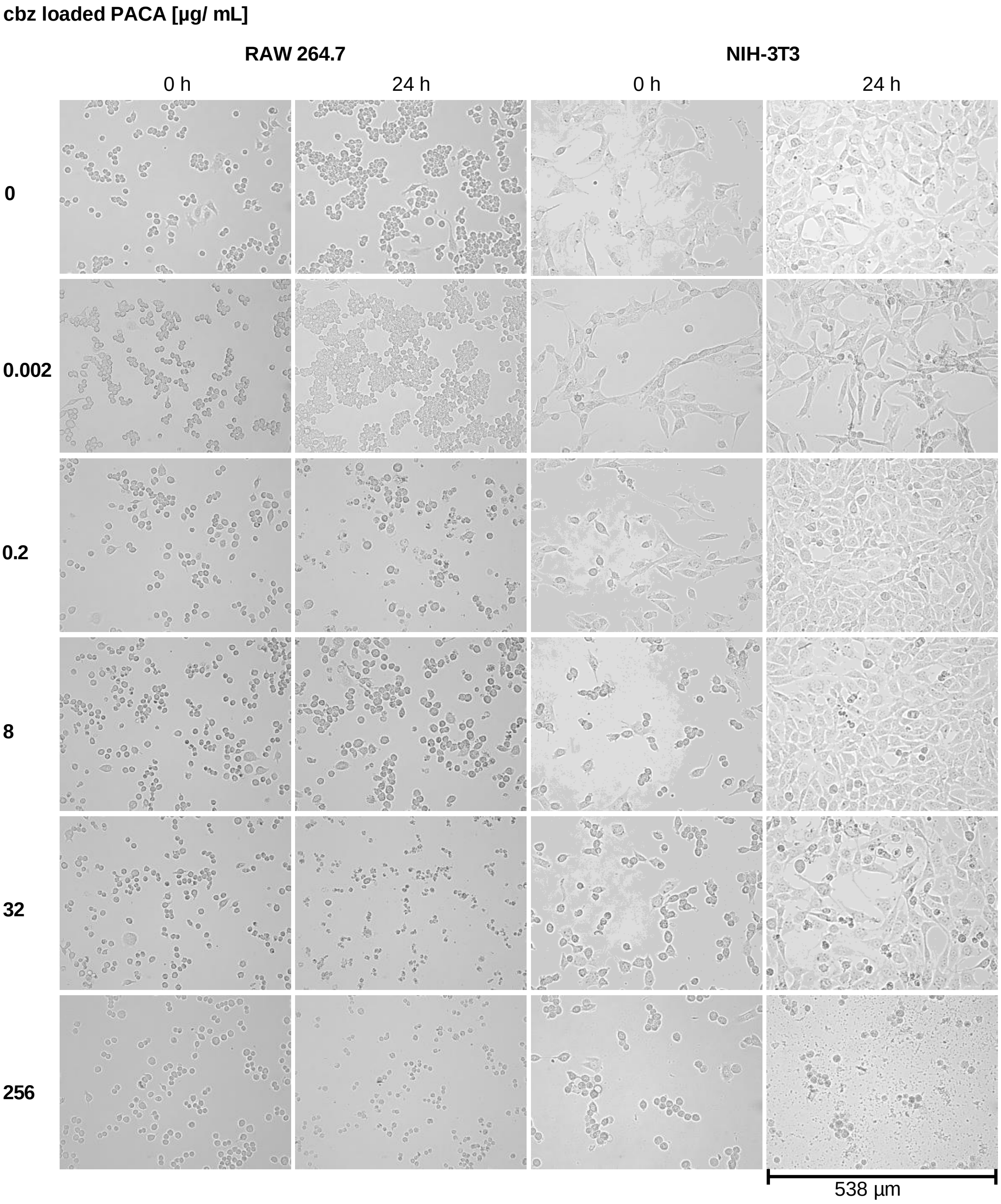

Figure S3. Bright-field images of RAW 264.7 macrophages and NIH-3T3 fibroblasts after incubation with cbz loaded PACA nanoparticles in five representatively selected concentrations (0.002, 0.2,8, 32 and 256 µg/mL) vs. cell culture medium controls (0 µg/mL) at time points t = 0 and t = 24 h. For both cell lines after incubation with cell culture medium control and 0.002 µg/mL of cbz loaded PACA nanoparticles viable cells were detected after 24 h. For 0.2 µg/mL cell debris could be observed for RAW 264.7. Macrophages incubated with 8 µg/mL of cbz loaded PACA show swollen but viable cell morphology after 24 h, and NIH-3T3 cells detached morphology at t = 0, but proliferated cells after 24 h. Cell debris is observed in both cell lines with 32 and 256 µg/mL of cbz loaded nanoparticles after 24 h.

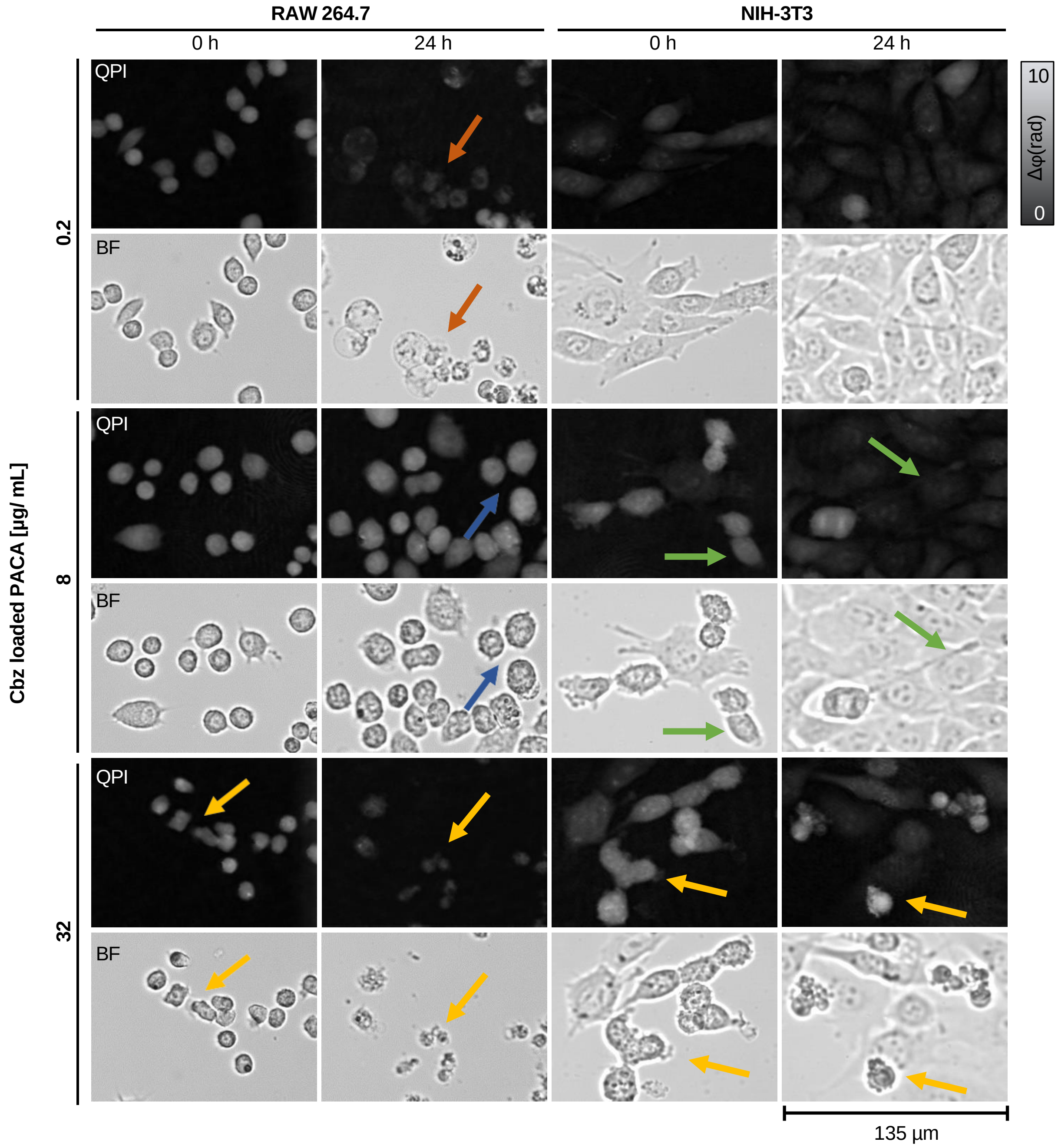

Figure S4. Enlarged areas of DHM-QPI images and bright-field images of RAW 264.7 macrophages and NIH-3T3 fibroblasts after incubation with cbz loaded PACA nanoparticles in three representatively selected concentrations (0.2, ,8 and 32) at time points t = 0 and t = 24 h. For 0.2 µg/mL of cbz loaded PACA nanoparticles cell debris was observed for RAW 264.7 macrophages after 24 h (orange colored arrows), and proliferated NIH-3T3 fibroblasts. At 8 µg/mL of cbz loaded PACA macrophages show after 24 h a swollen but viable cell morphology (blue colored arrows). In contrast, at this nanoparticle concentration, NIH-3T3 cells detached and cell debris is visible at t = 0 24 h, but after 24 h cells proliferated (green colored arrows). At 32 and 256 µg/mL of cbz loaded nanoparticles cell debris is observed after 24 h (yellow colored arrows).

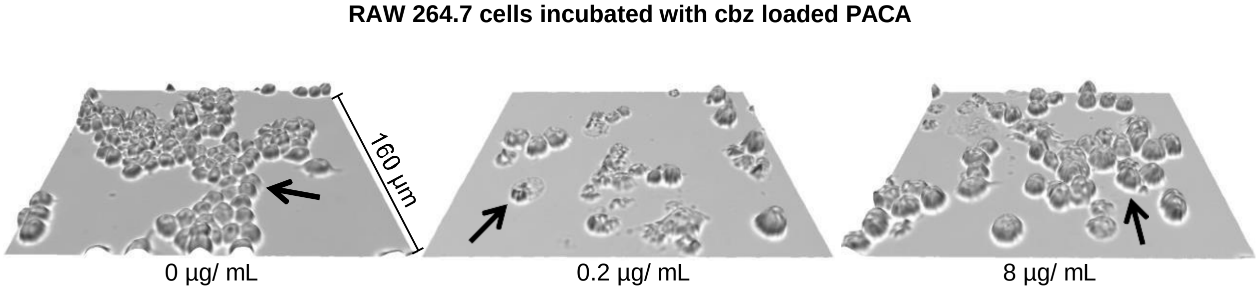

Figure S5. Pseudo 3D surface plots of representative DHM QPI image overlaid with the texture of correlatively recorded bright field images of RAW 264.7 macrophages 24 h after incubation with 0.2 and 8 µg/mL of cbz loaded PACA compared to medium control cells (0 µg/mL).

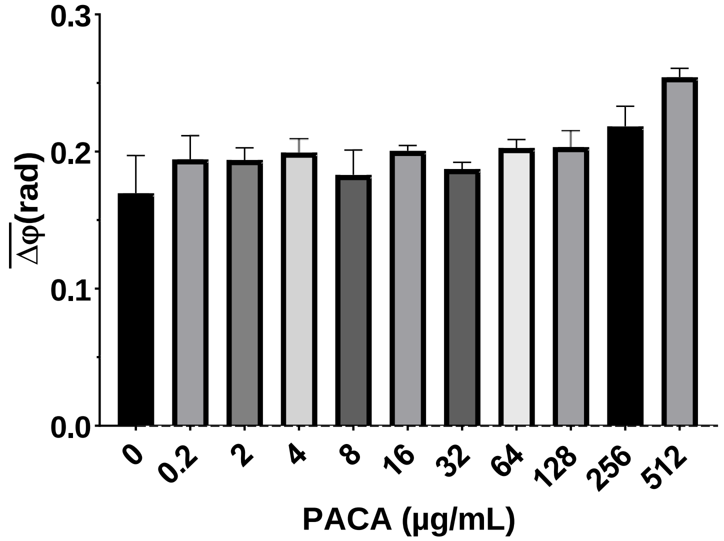

Figure S6. DHM QPI background noise level at different concentrations of unloaded PACA nanoparticles. For each nanoparticle concentration the mean phase contrast (Δφ) of three (n = 3) FOVs (405 x 538 µm) were measured after 24 h.

Information S1 Detailed analysis of non-monotonic dose-effect relationship of cbz-loaded PACA particles in this study and the literature

Comparable non-monotonic dose-effect observation as presented here has been described previously by Eder et al. where the cbz-loaded particles were tested with NIH-3T3 and RAW 264.7 cells in a cross-laboratory study [1]. Eder et al. observed an increase in cell viability and a decrease in toxicity for 8 µg/mL in all participating laboratories. Vrignaud et al. detected a regeneration of decreasing cell viability of human colon cells (HCT117) at 100 nM for cbz loaded PACA particles as well, before it re-increased again with ascending concentrations [2]. An explanation for the observed non-monotonic dose-effect relationship could be indicated by *in vivo* studies of Fan et al. and Palanza et al. [3, 4], They reported about non-monotonic dose response curves at high doses above the specified **N**o-**O**bserved-**A**dverse-**E**ffect **L**evel (NOAEL) from which could be concluded that the concentrations tested here were too high. This is in agreement with the results of the DHM (Figure 4D) and LDH (Figure 4F) assay which show that even the lowest concentrations already caused effects on cell proliferation or cell death. However, the absence of a significant effect for the RAW 264.7 cells and that a cell viability of 100 % is achieved for the WST-8 at the lowest concentrations (Figure 4E) contradicts this hypothesis. A review by Vandenberg et al. summarized that non-monotonic dose-effect relationships can occur even at low

concentrations [5]. To ensure that the concentrations tested in this study were not too high, our results are compared in the following with values for free cbz and PACA particles in the literature. Studies by Øverbye et al. demonstrated that the toxicity of PACAs loaded with cbz is very similar to that of free cbz. Øverbye et al. tested the toxicity of unloaded and cbz loaded PACA particles and free cbz for 72 h with nine different cell lines utilizing an MTT assay. EC_{50} values were not determined in the study, but inflection points were present between 1 and 10 nM of cbz for most of the investigated cell lines [6]. As cbz loaded PACA particles with 10 nM cbz correspond to 0.37–0.48 µg/mL of PACA materials [6], inflection points and mean EC_{50} values for PACAs loaded with cbz in our study should be in the range of the tested concentrations 0.002 and 0.2 µg/mL. A study of Vrignaud et al., investigated the toxicity of free cbz by a neutral red assay after 96 h on different cell lines and EC_{50} values between 0.004-0.041 µmol/ L were calculated [2]. This corresponds to the mentioned range (1 and 10 nM) by Øverbye et al. [6]. Further Chen et al. showed that the cytotoxic effects of free cbz also act in a time-dependent manner. In an MTT assay EC_{50} values between 20 and > 80 nM that were determined after 24 h for various cell lines, were much more lower than the values after 48 and 72 h [7]. This time-dependency of the results indicates that the EC_{50} values in our study should be higher than in Øverbye et al. and Vrignaud et al. With the assumption that the EC_{50} values for free cbz are higher after 24 h than after 48 and 72 h [7], and that 10 nM cbz corresponds to 0.37-0.48 µg/mL of PACA materials [6], we would expect for our study inflection points and EC_{50} values between 0.2 and 4 µg/mL. Therefore, it is suggested that the non-monotonic dose-response of cbz-loaded PACAs is due to the presence of multiple toxicity mechanisms leading to interaction or different types of cell death, as both the PACA particles and the cbz act on the cell in different ways. As both the PACA particles and the cbz act differently on the cell, for PACA particles, the degradation products are mainly responsible for their toxicity [8], whereas cbz inhibits microtubule disassembly [9, 10] which may lead to a superposition of the effects and to a different biodistribution and a different uptake into the cells [11]. This assumption is supported by the study by Szwed et al., in which various PACA particles were tested that differed only in their alkyl side chains and led to different toxicity mechanisms [12].

References

1. Eder, K.M.; Marzi, A.; Barroso, Á.; Ketelhut, S.; Kemper, B., and Schnekenburger, J., Label-free digital holographic microscopy for in vitro cytotoxic effect quantification of organic nanoparticles, *Cells,* **2022,** *11,* 4, p. 644.
2. Vrignaud, P.; Semiond, D.; Lejeune, P.; Bouchard, H.; Calvet, L.; Combeau, C.; Riou, J.F.; Commercon, A.; Lavelle, F., and Bissery, M.C., Preclinical antitumor activity of cabazitaxel, a semisynthetic taxane active in taxane-resistant tumors, *Clinical Cancer Research,* **2013,** *19,* 11, pp. 2973–2983.
3. Fan, F.; Wierda, D., and Rozman, K.K., Effects of 2,3,7,8-tetrachlorodibenzo-p-dioxin on humoral and cell-mediated immunity in Sprague-Dawley rats, *Toxicology* **1996,** *106,* 1-3, pp. 221-228.
4. Palanza, P.; Parmigiani, S., and vom Saal, F.S., Effects of prenatal exposure to low doses of diethylstilbestrol, o, p'DDT, and methoxychlor on postnatal growth and neurobehavioral development in male and female mice, *Hormones and Behavior,* **2001,** *40,* 2, pp. 252–265.
5. Vandenberg, L.N., Low-dose effects of hormones and endocrine disruptors, *Vitamins & hormones,* **2014,** *94,* pp. 129-165.
6. Øverbye, A.; Torgersen, M.L.; Sønstevold, T.; Iversen, T.G.; Mørch, Ý.; Skotland, T., and Sandvig, K., Cabazitaxel-loaded poly (alkyl cyanoacrylate) nanoparticles: toxicity and changes in the proteome of breast, colon and prostate cancer cells, *Nanotoxicology,* **2021,** *15,* 7, pp. 865-884.
7. Chen, R.; Cheng, Q.; Owusu-Ansah, K.G.; Chen, J.; Song, G.; Xie, H.; Zhou, L.; Xu, X.; Jiang, D., and S., Z., Cabazitaxel, a novel chemotherapeutic alternative for drug-resistant hepatocellular carcinoma, *American Journal of Cancer Research,* **2018,** *8,* 7, pp. 1297-1306.

8. Vauthier, C.; Dubernet, C.; Fattal, E.; Pinto-Alphandary, H., and Couvreur, P., Poly (alkylcyanoacrylates) as biodegradable materials for biomedical applications, *Advanced drug delivery reviews,* **2003,** *55,* 4, pp. 519-548.
9. Mita, A.C.; Figlin, R., and Mita, M.M., Cabazitaxel: More Than a New Taxane for Metastatic Castrate-Resistant Prostate Cancer?, *Clinical Cancer Research,* **2012,** *18,* 24, pp. 6574–6579.
10. Dumontet, C. and Jordan, M.A., Microtubule-binding agents: a dynamic field of cancer therapeutics, *Nature Reviews Drug Discovery,* **2010,** *9,* pp. 790-803.
11. Fusser, M.; Øverbye, A.; Pandya, A.D.; Mørch, Ý.; Borgos, S.E.; Kildal, W.; Snipstad, S.; Sulheim, E.; Fleten, K.G.; Askautrud, H.A.; Engebraaten, O.; Flatmark, K.; Iversen, T.G.; Sandvig, K.; Skotland, T., and Mælandsmo, G.M., Cabazitaxel-loaded Poly (2-ethylbutyl cyanoacrylate) nanoparticles improve treatment efficacy in a patient derived breast cancer xenograft, *Journal of Controlled Release,* **2019,** *293,* pp. 183-192.
12. Szwed, M.; Sønstevold, T.; Øverbye, A.; Engedal, N.; Grallert, B.; Mørch, Ý.; Sulheim, E.; Iversen, T.G.; Skotland, T.; Sandvig, K., and Torgersen, M.L., Small Variations in Nanoparticle Structure Dictate Differential Cellular Stress Responses and Mode of Cell Death, *Nanotoxicology,* **2019,** *13,* pp. 1-22.
